# Time spent in prior hospital stay and outcomes for ventilator patients in long-term acute care hospitals

**DOI:** 10.1186/s12890-021-01454-1

**Published:** 2021-03-24

**Authors:** Berna Demiralp, Lane Koenig, Jing Xu, Samuel Soltoff, John Votto

**Affiliations:** 1 KNG Health Consulting, LLC, 15245 Shady Grove Road, # 365, Rockville, MD 20850 USA; 2 Hospital for Special Care, 2150 Corbin Ave, New Britain, CT 06053 USA

**Keywords:** Ventilator weaning, Mortality, Length of stay

## Abstract

**Background:**

Long-term acute care hospitals (LTACHs) treat mechanical ventilator patients who are difficult to wean and expected to be on mechanical ventilator for a prolonged period. However, there are varying views on who should be transferred to LTACHs and when they should be transferred. The purpose of this study is to assess the relationship between length of stay in a short-term acute care hospital (STACH) after endotracheal intubation (time to LTACH) and weaning success and mortality for ventilated patients discharged to an LTACH.

**Methods:**

Using 2014–2015 Medicare claims and assessment data, we identified patients who had an endotracheal intubation in STACH and transferred to an LTACH with prolonged mechanical ventilation (defined as 96 or more consecutive hours on a ventilator). We controlled for age, gender, STACH stay procedures and diagnoses, Elixhauser comorbid conditions, and LTACH quality characteristics. We used instrumental variable estimation to account for unobserved patient and provider characteristics.

**Results:**

The study cohort included 13,622 LTACH cases with median time to LTACH of 18 days. The unadjusted ventilator weaning rate at LTACH was 51.7%, and unadjusted 90-day mortality rate was 43.7%. An additional day spent in STACH after intubation is associated with 11.6% reduction in the odds of weaning, representing a 2.5 percentage point reduction in weaning rate at 18 days post endotracheal intubation. We found no statistically significant relationship between time to LTACH and the odds of 90-day mortality.

**Conclusions:**

Discharging ventilated patients earlier from STACH to LTACH is associated with higher weaning probability for LTACH patients on prolonged mechanical ventilation. Our findings suggest that delaying ventilated patients’ discharge to LTACH may negatively influence the patients’ chances of being weaned from the ventilator.

**Supplementary Information:**

The online version contains supplementary material available at 10.1186/s12890-021-01454-1.

## Background

The number of critically ill patients receiving ventilator care has grown considerably over the last several years [1]. While the use of ventilator support has extended the life of patients, those who survive face poor prognosis, with high 1-year mortality rates [2, 3]. A key step to maximize the recovery of these patients is liberating them from the ventilator. However, only about half of patients are liberated from mechanical ventilation upon hospital discharge [3].

In the U.S., many critically ill patients on ventilators are discharged to long-term acute care hospitals (LTACHs) to wean off the ventilator. These specialty hospitals, of which there are fewer than 400 in the nation, have an average length of stay of over 25 days and care for critically ill patients requiring an extended inpatient hospital stay [4]. In 2017, roughly 44% of Medicare fee-for-service (FFS) patients on prolonged mechanical ventilation (PMV) (defined as 96 or more consecutive hours on a ventilator) and discharged alive from a short-term acute care hospital (STACH) to a post-acute care setting were admitted to an LTACH, compared to 3% of all hospitalized Medicare FFS patients.^1^

To support discharge decisions, many hospitals and payers use proprietary or commercial clinical guidelines to help in patient placement assessments. These guidelines provide criteria for when it is appropriate for a ventilated patient to be admitted to an LTACH. In 2005, a consensus statement from the National Association for Medical Direction of Respiratory Care (NAMDRC) recommended that prolonged mechanical ventilation be defined as the need for 21 consecutive days or longer on mechanical ventilation for at least 6 h a day (abbreviated “21-day requirement”) [5]. Based on this recommendation, private health plans covering Medicare beneficiaries (Medicare Advantage plans) often cite failure to meet the 21-day requirement as a key reason for prior-authorization denials for admission to an LTACH.

In this study, we assess the relationship between length of stay in a STACH after endotracheal intubation and mortality and weaning success for ventilated patients discharged to an LTACH. Earlier discharged to an LTACH for ventilator weaning services may have two conflicting effects. First, patients transferred earlier may experience improved outcomes due to receipt of specialized ventilator weaning services at LTACHs. On the other hand, if early transfer patients are less stable, requiring a level of care only available in an intensive care unit (ICU), discharging ventilated patients too early to an LTACH may negatively affect outcomes. We use an instrumental variable approach to conduct the analysis in recognition that STACH discharge decisions may be influenced by patient complexity and financial consideration not readily observed in administrative data.

## Methodology

We constructed our study cohort, explanatory variables, and outcomes using 2014–2015 Medicare claims and assessment data. The Medicare claims data were used to study services provided by STACHs, LTACHs, inpatient rehabilitation facilities (IRFs), skilled nursing facilities (SNFs), and home health agencies (HHAs). We used assessment data from the Minimum Data Set to analyze stays in nursing homes (NHs). We obtained dates of admissions and discharges, patient demographic information, and clinical diagnosis codes and procedure codes for each hospitalization and LTACH transfer. We used LTACH Compare data available on the Centers for Medicare & Medicaid (CMS) website to obtain LTACH characteristics on quality during 2014 and 2015. Ethical & Independent Review Services determined that a waiver from the requirement to obtain HIPPA authorization was justified for this research project.

Our study population consisted of Medicare beneficiaries who were aged 65 or older, discharged from a STACH between January 1, 2014 and June 30, 2015, and, subsequently, admitted to an LTACH. We limited the analytic sample to Medicare beneficiaries who: (1) received a tracheostomy (ICD 9 procedure codes: "311","312","3121","3129") at the STACH; (2) were on prolonged mechanical ventilation at the STACH and LTACH; (3) spent at least one day in an ICU during the STACH stay; (4) were admitted to an LTACH same or next day after discharge from STACH; and (5) died in the LTACH or were discharged from the LTACH to another hospital, IRF, SNF, NH, hospice, or home with or without home health care.

Our sample exclusions included: (1) beneficiaries who had Medicare Advantage coverage during the month of LTACH stay and the following month; (2) beneficiaries who did not have continuous Part A coverage during the month of LTACH stay and the following month; (3) beneficiaries who were admitted to a STACH for the non-surgical treatment of cancer; (4) beneficiaries who had conditions that were not suitable to wean, such as quadriplegia, or amyotrophic lateral sclerosis; (5) beneficiaries who discharged from a STACH or LTACH against medical advice; (6) beneficiaries who exhausted Medicare benefits during a STACH or LTACH stay; and (7) beneficiaries who had overlapping stays in a STACH or LTACH based on dates of admission and discharge in claims or had a missing admission or discharge date. We also excluded outlier cases in terms of days from intubation at the STACH to LTACH transfer (“time to LTACH”), defined as those with days outside of the 1st and 99th percentiles of the time to LTACH distribution, from the analysis.

We examined two clinical outcomes: ventilator weaning during LTACH stay and mortality. A patient was considered to have weaned off the ventilator if he/she was discharged alive from the LTACH and not on mechanical ventilation in the next observed setting after discharge from the LTACH. We measured mortality within 90 days starting at day of LTACH admission. We performed logistic regression to estimate the outcomes.

The key explanatory variable in the analysis was “time to LTACH” (TTL), which was defined as the length of time between endotracheal intubation (ICD 9 procedure code: “9604”) in the STACH to discharge from STACH. We adapted a risk-adjustment model developed by Kahn et al. to examine in-LTACH mortality for a ventilated population [6]. In addition to TTL, we obtained clinical information from the STACH stay and constructed risk-adjustment variables based on the model developed by Kahn et al. for 90-day mortality rates among patients admitted to LTACHs for ventilator weaning. Patient characteristics included: age, gender, STACH stay procedures and diagnoses (coronary artery bypass graft (CABG), dialysis, hypotension, percutaneous transluminal coronary angioplasty (PTCA), stroke, thrombocytopenia, trauma, and valve replacement), and Elixhauser comorbid conditions during STACH stay. We also controlled for two CMS LTACH quality measures obtained from LTACH Compare: 30-day all-cause unplanned readmission rate and percent of patients with new or worsened pressure ulcers [7]. We added these quality proxy measures, which are readily available from CMS and validated, to control for differences in patient outcomes that may be related to LTACH quality and correlated with TTL.

TTL may be correlated with factors unobservable from claims and assessment data, such as STACH preferences, STACH quality, payer differences, and patient severity. The direction of the correlation is unclear as factors determining TTL may be complex. For example, patients who stay longer in the acute care hospital may be sicker or it may reflect STACH physician preference to keep the patient under his or her direct care longer. On the other hand, patients who have shorter lengths of stay may be less acutely ill or debilitated or may be discharged sooner by hospitals to limit financial losses on complex patients. We accounted for the potential endogeneity of TTL by using instrumental variable (IV) estimation. The IV used in the analysis was the residual from a linear regression of LTACH daily census of all patients on LTACH daily census of ventilator patients. By constructing the IV based on this regression residual, our IV is not correlated with LTACH daily census of ventilator patients, which may be correlated with outcomes if there is a volume-quality relationship for treating ventilator patients.

Since the models for outcomes were non-linear, we used two-stage residual inclusion (2SRI) estimation to conduct the IV analysis [8]. In the first stage, we used TTL as the dependent variable, and we included IV and all the other covariates as independent variables. The model we used was generalized linear model (GLM) with a Gaussian distribution, a log-link function and robust standard errors. We obtained the residuals from the GLM to include in the second stage. Specifically, in the second stage, we use logistic regression, in which the outcome variables were weaning or 90-day mortality, and the explanatory variables included TTL, residual from the first stage GLM, age categories, gender, and all the clinical conditions and comorbidities. We used bootstrapping (500 replications) to approximate the asymptotically correct standard errors.

For the weaning outcome, we conducted two sets of sensitivity analysis. In the first set of sensitivity analysis, we only considered a patient to have weaned if he/she met the original definition (i.e., patient discharged alive from the LTACH and not on mechanical ventilation in the next setting after LTACH discharge) and the patient was alive in the next setting for at least 7 days. In the second set of sensitivity analysis, we excluded patients who went to hospice after LTACH discharge from our analytic sample.

For the logistic regression and the 2SRI IV analysis, we calculated the odds ratio and marginal effects for TTL. We considered a *p* value less than 0.05 to be statistically significant. All the analyses were performed with STATA 16.0 (StataCorp LLC, College Station, Texas).

## Results

Our study cohort included 13,622 Medicare beneficiaries who had an endotracheal intubation in a STACH and were discharged to an LTACH between January 1, 2014 and June 30, 2015 (Table 1). Average length of stay in the STACH prior to admission to the LTACH was 24.0 days, and the average number of days between endotracheal intubation and discharge from STACH was 19.6 days. The number of days to LTACH after endotracheal intubation had a median of 18 and interquartile range of [13, 24]. Some of the common diagnoses in the study cohort included congestive heart failure (45.8%), cardiac arrhythmias (52.7%), chronic pulmonary disease (48.3%), and fluid and electrolyte disorders (78.7%).

**Table 1:** Patient characteristics

	All	Weaned at LTACH	Not Weaned at LTACH	Alive in 90 Days	Expired in 90 Days
No. of Cases	13,622	7046	6576	7668	5954
**Time to LTACH**					
Mean	19.6	19.1	20.2	19.2	20.2
25th percentile	13.0	13.0	14.0	13.0	14.0
Median	18.0	17.0	18.0	18.0	18.0
75th percentile	24.0	23.0	25.0	23.0	25.0
Age	70.6	68.7	72.7	68.6	73.2
Male	50.4%	50.2%	50.7%	49.3%	51.9%
Prior STACH LOS	24.0	23.2	24.9	23.3	25.0
**Prior STACH Procedures and Diagnoses**					
Dialysis	15.2%	13.2%	17.5%	12.4%	18.9%
PTCA	1.6%	1.4%	1.7%	1.6%	1.5%
CABG	3.9%	3.8%	4.1%	3.6%	4.4%
Valve Replacement	2.8%	2.6%	3.0%	2.4%	3.3%
Hypotension	10.9%	11.2%	10.6%	11.1%	10.6%
Thrombocytopenia	15.9%	15.5%	16.3%	15.0%	17.0%
Stroke	13.5%	15.0%	12.0%	14.1%	12.7%
Trauma	9.8%	10.7%	8.8%	10.2%	9.3%
**Exlihauser Comorbidity**					
Congestive Heart Failure	45.8%	40.5%	51.4%	41.6%	51.1%
Cardiac Arrhythmias	52.7%	48.6%	57.0%	49.3%	57.0%
Valvular Disease	12.3%	10.6%	14.2%	10.7%	14.4%
Pulmonary Circulation Disorders	15.4%	13.6%	17.2%	14.6%	16.4%
Peripheral Vascular Disorders	12.1%	11.5%	12.7%	11.2%	13.3%
Hypertension, Uncomplicated	39.3%	42.7%	35.7%	43.0%	34.7%
Paralysis	8.9%	10.3%	7.5%	10.1%	7.4%
Other Neurological Disorders	44.3%	44.6%	44.1%	44.7%	43.8%
Chronic Pulmonary Disease	48.3%	45.2%	51.5%	47.3%	49.5%
Diabetes, Uncomplicated	30.0%	30.3%	29.7%	29.7%	30.3%
Diabetes, Complicated	8.9%	8.3%	9.6%	8.2%	9.8%
Hypothyroidism	13.9%	13.8%	14.0%	13.3%	14.5%
Renal Failure	30.2%	26.3%	34.3%	25.9%	35.7%
Liver Disease	9.1%	9.0%	9.3%	8.7%	9.7%
Peptic Ulcer Disease Excluding Bleeding	1.7%	1.7%	1.7%	1.8%	1.6%
AIDS/HIV	0.4%	0.4%	0.5%	0.4%	0.5%
Lymphoma	1.1%	1.0%	1.2%	0.8%	1.4%
Metastatic Cancer	1.4%	0.9%	2.1%	0.8%	2.3%
Solid Tumor Without Metastasis	3.7%	2.6%	4.9%	2.4%	5.4%
Rheumatoid Arthritis/Collagen Vascular	3.7%	3.9%	3.4%	3.8%	3.5%
Coagulopathy	19.9%	19.3%	20.5%	18.6%	21.5%
Obesity	21.3%	23.4%	19.0%	24.0%	17.7%
Weight Loss	42.8%	40.9%	44.8%	40.5%	45.7%
Fluid and Electrolyte Disorders	78.7%	78.6%	78.9%	78.4%	79.2%
Blood Loss Anemia	1.8%	1.8%	1.7%	1.7%	1.8%
Deficiency Anemia	4.1%	4.5%	3.7%	4.4%	3.7%
Alcohol Abuse	5.8%	6.9%	4.6%	6.7%	4.7%
Drug Abuse	3.2%	4.3%	2.0%	4.1%	2.1%
Psychoses	3.1%	3.8%	2.4%	3.6%	2.5%
Depression	11.6%	13.1%	10.0%	13.3%	9.5%
Hypertension, Complicated	28.4%	25.0%	32.1%	24.4%	33.5%
Number of Elixhauser comorbidities (mean)	6.0	5.9	6.2	5.9	6.2

All of the variables in Table 1, except for the prior STACH LOS and the number of Elixhauser comorbidities, were included in the risk adjustment model. Age categories were included as indicator variables (age categories: <  = 70,71–75,76–80,81 +)

Patients who were weaned off the ventilator at the LTACH, on average, were younger (68.7 vs. 72.7) and had shorter time in STACH between endotracheal intubation and discharge to LTACH (19.1 days vs. 20.2 days). In addition, patients who weaned were less likely to have 19 of the 31 Elixhauser comorbidities examined. Similar to patients who weaned, patients who survived the 90-day period post LTACH admission were younger (68.6 vs. 73.2) with shorter TTL (19.2 vs. 20.2 days). They were also less likely to have 20 of the 31 Elixhauser comorbidities examined.

The unadjusted ventilator weaning rate at LTACHs in our study cohort is 51.7%, indicating that more than half of the patients in our study weaned from the ventilator at the LTACH and were discharged alive (Table 2). Weaning rates were lower in our sensitivity analyses. Unadjusted weaning rate was 47.1 and 48.0% when weaned patients were required to remain alive for 7 days following discharge from LTACH or when excluding patients discharged from LTACH to hospice, respectively. The 90-day mortality rate was 43.7 percent. The correlation coefficient between TTL and ventilator weaning ranged between − 0.0623 and -0.0652 (depending on weaning definition), and the correlation coefficient between TTL and 90-day mortality was 0.0570.

**Table 2:** Unadjusted outcomes

	Overall sample size	Outcome rate	Correlation coefficient between outcome and time to LTACH
Weaned at LTACH	13,622	0.517	− 0.0629
Weaned at LTACH and alive for at least 7 days after discharge	13,622	0.471	− 0.0623
Weaned at LTACH, excluding patients discharged to hospice	13,202	0.480	− 0.0652
90-day Mortality	13,622	0.437	0.0570

Next, we examined the relationship between TTL and outcomes (probability of weaning and mortality) using logistic regression. Without controlling for any patient characteristics, each additional day in STACH after endotracheal intubation is associated with a 1.4% decrease in the odds of weaning from the ventilator in the LTACH (Table 3). When we control for patient clinical characteristics, each additional day in STACH after endotracheal intubation is associated with a 1% reduction in the odds of weaning off the ventilator at the LTACH. This decrease from 1.4 to 1.0% suggests that patient clinical characteristics accounts for some of the negative relationship between time to LTACH and weaning probability. This relationship holds when weaned patients are limited to those who not only wean from the ventilator but also remain alive for at least 7 days after discharge from LTACH and when the study cohort excludes patients who were discharged from LTACH to hospice.

**Table 3:** Relationship between Time to LTACH and Outcomes

Outcomes	Univariate Logistic Regression		Multivariate Logistic Regression		Instrumental Variable Regression	
	Odds ratio	*P* value	Odds ratio	*P* value	Odds ratio	*P* value
Weaned at LTACH	0.986	< 0.001	0.990	< 0.001	0.884	0.005
Weaned at LTACH and alive for at least 7 days after discharge	0.986	< 0.001	0.990	< 0.001	0.857	0.001
Weaned at LTACH, excluding patients discharged to hospice	0.986	< 0.001	0.990	< 0.001	0.866	0.001
90-day Mortality	1.013	< 0.001	1.007	0.001	1.046	0.327

Our estimation of ventilator weaning probability using IV yielded that an additional day spent in STACH after endotracheal intubation is associated with 11.6% reduction in the odds of weaning off the ventilator in LTACH (Table 3). If we define ventilator weaning as weaning off of ventilator and being alive for at least 7 days post discharge from LTACH, the odds of weaning decreases by 14.3% for each additional day in STACH after endotracheal intubation. When we exclude patients discharged from LTACH to hospice, we find that an additional day in STACH is associated with a 13.4% reduction in odds of weaning off ventilator at LTACH.

Because the models are non-linear, the effects of TTL on outcomes vary at different points in the TTL distribution. To better understand the relationship between TTL and the probability of weaning from ventilator at LTACH, we examined the average predicted probability of weaning if patients were discharged to an LTACH at 13, 18, and 24 days after endotracheal intubation, which correspond to the 25^th^, 50^th^, and 75^th^ percentile of the TTL distribution in the study cohort (Table 4). Based on instrumental variable estimation results, the average predicted probability of weaning is 54.5% at the median time to LTACH of 18 days post endotracheal intubation. The average adjusted probability of weaning decreases by 2.5 percentage points to 52.0% at 19 days. The average adjusted probability of being weaned decreases by 27 percentage points from 66.7 to 39.7% when time to LTACH increases from 13 to 24 days. We consistently observed reductions in the average adjusted probability of weaning with an increase in time to LTACH under alternative definitions of weaning. For example, when weaned patients are defined as those who wean from the ventilator at LTACH and remain alive for at least 7 days post discharge from LTACH, the probability of weaning decreases by 31 percentage points from 65.1 to 34.1% between 13 and 24 TTL days. When the study cohort excludes patients discharged to hospice, the average predicted probability of weaning decreases by 29.7 percentage points from 65.1 to 35.4% between 13 and 24 TTL days.

**Table 4:** Average Adjusted Outcomes at Select Values of Time to LTACH

	Adjusted probability of being weaned at LTACH		Adjusted probability of being weaned at LTACH and alive for at least 7 days after discharge from LTACH		Adjusted probability of being weaned at LTACH, excluding patients discharged to hospice	
Time to LTACH	Logistic Regression without IV	IV Estimation	Logistic Regression without IV	IV Estimation	Logistic Regression without IV	IV Estimation
13 days (25th percentile)	0.534	0.667	0.486	0.651	0.496	0.651
17 days	0.524	0.570	0.477	0.537	0.486	0.542
18 days (median)	0.521	0.545	0.475	0.507	0.484	0.514
19 days	0.519	0.520	0.473	0.478	0.481	0.487
24 days (75th percentile)	0.507	0.397	0.461	0.341	0.469	0.354

Without controlling for patient characteristics, we found that an additional day in a STACH before discharge to an LTACH is associated with 1.3% increase in the odds of 90-day mortality among LTACH patients on PMV (OR = 1.013, *p* value < 0.001) (Table 3). Controlling for patient clinical characteristics, an additional TTL day is associated with 0.7% increase in the odds of 90-day mortality (OR = 1.007, *p* value = 0.001). When we used instrumental variable estimation, we found no statistically significant relationship between TTL and the odds of 90-day mortality after admission to LTACH (OR = 1.046, *p* value = 0.327).

We conducted diagnostic tests on the instrumental variable to assess its validity. We found that the F-statistic on the excluded instrument in the first-stage regression was 23.34 (25.43 when patients discharged to hospice are excluded) (See Additional file 1: Table S1). This F-statistic is greater than the Stock-Yogo critical value of 16.38, supporting that the instrument is strongly related to TTL. There is no test for the exogeneity of the instrument when there is only one instrumental variable for the one endogenous variable. Although we cannot assess the relationship between the instrument and unobservable characteristics, we examined the relationship between instrument and observable characteristics. Specifically, we divided the TTL into quartiles and assessed whether the average patient characteristics varied across the quartiles. We provide this information in Supplemental Material (Additional file 1: Table S2).

## Discussion

The number of patients on prolonged mechanical ventilation has increased over the recent decades due to advances in intensive care medicine. For example, in the US, rate of endotracheal intubation in the adult population more than doubled between 1993 and 2012 [1]. In the U.S., LTACHs are a common care setting for patients to attempt to wean, after an extended period on a ventilator. However, our understanding of the outcomes for these chronically critically ill patients and how to improve their outcomes has lagged, resulting in an important knowledge gap [3, 9].

In this study, we examine ventilator weaning and mortality rates among Medicare beneficiaries treated with PMV in LTACHs after receiving an endotracheal intubation in a STACH. Our findings contribute to our understanding of both the outcomes for patients with PMV and the determinants of their outcomes. We found that about 51.7% of patients in our study population weaned from the ventilator at LTACH; 47.1% of the patients weaned and remained alive for at least 7 days after discharge from LTACH. We also found that more than half (56.3%) of the study population was alive within 90-days of being admitted to the LTACH. The median time between endotracheal intubation and admission to LTACH was 18 days, with an interquartile range of 11 days (13 to 24). Our study revealed that the variation in time spent in STACH after endotracheal intubation is associated with the patient’s probability of being weaned during the subsequent LTACH stay. Patients discharged to LTACHs earlier have higher risk-adjusted odds of weaning. In particular, we found that an additional day spent in STACH after endotracheal intubation reduces the odds of weaning by 11.6% for patients discharged to LTACHs. This represents a reduction of weaning probability from 54.5 to 52.0% for patients at median TTL of 18 days. We found no statistically significant relationship between TTL and 90-day mortality.

Our findings are consistent with previous research documenting that patients on PMV have poor prognosis. In their meta-analysis of prior research findings, Damuth and co-authors reported pooled one-year mortality rate of 73% and ventilator weaning rate of 47% based on studies that examine outcomes for ventilator patients treated in post-acute hospitals [3]. Compared to previous studies of LTACH patients included in Damuth et al.’s meta-analysis, our study has the largest sample size (13,622 cases) as it includes Medicare FFS beneficiaries patients treated in all LTACHs in the US during the study period. The largest previous study of LTACH patients by Scheinhorn et al., examining ventilator weaning, included 1,419 patients treated in 23 LTACHs and reported a ventilator weaning rate of 54.1% [10]. The higher weaning rate in Scheinhorn et al. study as compared to 51.7% in our study may be partially due to differences in population exclusions between the two studies. Scheinhorn et al. excluded patients admitted to LTACH for end-of-life care (terminal weaning) and for home ventilator training whereas our study did not implement these exclusions.

Our study has limitations that should be considered in reviewing our results. First, due to data limitation, we identify ventilator weaning based on Medicare claims and assessment data in the next care setting following the LTACH stay instead of assessment data from the LTACH. Although the ventilator weaning rate in our study falls within benchmarks from prior studies, including the Ventilation Outcomes Study, the measurement of ventilator weaning based on next setting may have limited accuracy in measuring weaning at LTACH. Second, although we present supporting evidence on the exogeneity of our instrumental variable, we are unable to confirm its exogeneity due to lack of such a test in the case of a single instrument. Third, patients discharged home from LTACH are included in the weaned group although we do not have data on these patients’ ventilator use after discharge from LTACH. Since some of the patients discharged home may be on hospice or end-of-life care, we used alternative measures of weaning outcomes that include patients who die soon after LTACH discharge in the non-weaned group. Finally, our analysis is based on patients who are enrolled in traditional Medicare. Therefore, our findings may not be generalizable to patients covered by Medicare Advantage or other commercial plans.

LTACHs are an important care setting for ventilated patients. In 2013, half of all LTACHs had at least 21.7% of their patients on a ventilator [6]. The introduction of patient criteria in Medicare LTACH payment policy starting in 2016, which reduced payments for cases that did not spend at least 3 days in an intensive care unit prior to admission, has increased the proportion of LTACH patients on a ventilator, further underscoring the importance of LTACHs as specialized treatment facilities for ventilated patients [11]. The number of patients on PMV is likely to continue to grow in the coming years, as a result of continued treatment improvements that keep critical ill patients alive longer and the aging of the U.S. population.

By providing specialized ventilator weaning units, LTACHs may offer benefits to patients and, more broadly, the healthcare system. Shorter TTL may improve weaning outcomes in LTACHs due to: (1) use and earlier implementation of weaning protocols; (2) use and earlier implementation of a rehabilitation model of care, including early and frequent ambulation; and (3) use of multidisciplinary team approach of care which brings together nurses, pharmacists, physicians, nutritionists, pastoral care, social workers and often psychologists. Rak et al. found that high-performing LTACHs in the treatment of ventilated patients differed from lower performers in the promotion of interdisciplinary communication and coordination through, for example, the use of care protocols and interdisciplinary team [12]. Short-term acute care hospitals may be less able to focus on the specific needs of these patients in their intensive care units [13]. In addition, studies have found that hospital volume of ventilated patients may be positively correlated with survival [14–17]. LTACHs may also relieve pressure on hospital intensive care units, reducing over-crowding which may improve outcomes [18].

Even if some ventilated patients may benefit from LTACH care, not all patients on a ventilator in a short-term acute care hospital may be appropriate to receive care in an LTACH. Some patients may be able to wean off a ventilator at the STACH, while others may have such poor prognosis that weaning is highly unlikely. In these cases, transfer to an LTACH may increase healthcare spending with little measurable benefits. As a result, who should be transferred to an LTACH for ventilator weaning care and the timing of the transfer are key clinical questions in the treatment of patients with PMV requiring investigation.

## Conclusions

Our findings have important implications for the treatment of chronically critically ill patients on prolonged mechanical ventilation. While LTACHs may offer specialized services and support to treat mechanical ventilator patients who are difficult to wean and expected to be on mechanical ventilator for a prolonged period of time, identifying such patients prospectively based on clinical characteristics has proven to be difficult due to lack of clinical evidence. Partially due to this difficulty in identifying candidates for LTACH care, clinical guidelines have emphasized a “test of time”, such as minimum 21 days in a STACH, before transfer to an LTACH. Our findings show that timing of patients’ discharge to LTACH may negatively influence the patients’ chances of weaning from the ventilator. Earlier access to LTACH care is associated with higher weaning probability for LTACH patients, suggesting patients may benefit from earlier discharge to LTACH and raising questions as to the clinical value of “test of time” approaches to LTACH transfer.

The current COVID-19 pandemic and the widespread use of mechanical ventilation in the treatment of COVID-19 patients has put a spotlight on the outcomes and treatment alternatives of patients on prolonged mechanical ventilation. In preparation for the surge of COVID-19 patients, some have argued that LTACHs can play an important role in freeing up capacity in STACHs by caring for non-COVID-19 patients and serving as overflow setting for COVID-19 patients [19]. Our findings suggest that discharging non-COVID-19 PMV patients earlier to LTACHs during a potential surge of COVID-19 patients may not bring about trade-offs in patient outcomes. On the contrary, earlier discharge to LTACHs may increase the odds of ventilator weaning for these patients. In addition, while available data are limited, studies indicate that some patients with critical illness from COVID-19 will be intubated and on prolonged ventilation. Some of these patients may face challenges in weaning from the ventilator, and LTACHs are likely to treat some of these patients. An important question for future research is to better understand the appropriate care and care setting for COVID-19 patients on PMV.

## Supplementary Information

**Additional file 1.** Instrumental Variable Diagnostics.

## Data Availability

The data used in this study was obtained from Centers for Medicare & Medicaid Services (CMS) under a Data Use Agreement (DUA), and therefore, they are not publicly available but may be requested by submitting a DUA request to CMS.

## Abbreviations

FFS: Fee-for-service
GLM: Generalized linear model
HHA: Home health agency
ICU: Intensive care unit
IRF: Inpatient rehabilitation facility
IV: Instrumental variable
LTACH: Long-term acute care hospital
LOS: Length of stay
NAMDRC: National Association for Medical Direction of Respiratory Care
NH: Nursing home
PMV: Prolonged-mechanical ventilation
SNF: Skilled nursing facility
STACH: Short-term acute care hospital
TTL: Time between endotracheal intubation in the STACH and discharge from STACH to LTACH.
2SRI: Two-stage residual inclusion

## Footnotes

1 Based on authors’ analysis of Medicare FFS claims data.
