# Supplementary material for: Time spent in prior hospital stay and outcomes for ventilator patients in long-term acute care hospitals
Source: BMC Pulm Med. 2021 Mar 24;21:104. doi: 10.1186/s12890-021-01454-1 (PMC7990091; doi:10.1186/s12890-021-01454-1)
Supplement: Supplementary file 1 — Additional file 1. Instrumental Variable Diagnostics. [file 12890_2021_1454_MOESM1_ESM.docx]

**TIME SPENT IN PRIOR HOSPITAL STAY AND OUTCOMES FOR VENTILATOR PATIENTS IN LONG-TERM ACUTE CARE HOSPITALS**

**SUPPLEMENTARY MATERIAL**

**Berna Demiralp**

KNG Health Consulting, LLC

15245 Shady Grove Road, # 365

Rockville, MD 20850

**Lane Koenig**

KNG Health Consulting, LLC

15245 Shady Grove Road, # 365

Rockville, MD 20850

**Jing Xu**

KNG Health Consulting, LLC

15245 Shady Grove Road, # 365

Rockville, MD 20850

**Samuel Soltoff**

KNG Health Consulting, LLC

15245 Shady Grove Road, # 365

Rockville, MD 20850

**John Votto**

Hospital for Special Care

2150 Corbin Ave
New Britain, CT 06053

**Corresponding Author:** Lane Koenig

KNG Health Consulting, LLC

15245 Shady Grove Road, #365, Rockville, MD 20850

Phone: 240 403 0154 x301, Fax: (301) 339-8436

Lane.koenig@knghealth.com

**SUPPLEMENTARY MATERIAL**

**Table S1. First Stage of Instrumental Variable Regression**

|  | **Overall cohort (N=13,622)** | **Cohort, excluding cases discharged from LTACH to hospice (N=13,202)** |
| --- | --- | --- |
| Coefficient on Instrument (std error in parentheses) | 0.119 (0.025) | 0.126 (0.025) |
| F-statistic on instrument | 23.34 | 25.43 |

**Table S2. Patient Characteristics by Quartiles of Instrumental Variable (IV)**

|  | **IV** | | | |
| --- | --- | --- | --- | --- |
|  | Quartile 1 | Quartile 2 | Quartile 3 | Quartile 4 |
| Age |  |  |  |  |
| 70 and Under | 46.3% | 46.9% | 46.4% | 44.8% |
| 71 to 75 | 17.9% | 18.0% | 18.6% | 18.3% |
| 76 to 80 | 15.5% | 17.0% | 15.3% | 15.7% |
| 81 and Above | 20.4% | 18.2% | 19.7% | 21.2% |
| Male | 50.3% | 50.5% | 50.7% | 50.2% |
| *Prior STCH Procedures and Diagnoses* | | |  |  |
| Dialysis | 14.2% | 15.0% | 14.9% | 16.9% |
| PTCA | 1.3% | 1.8% | 1.9% | 1.3% |
| CABG | 4.3% | 4.4% | 3.8% | 3.3% |
| Valve Replacement | 2.7% | 3.0% | 2.7% | 2.7% |
| Hypotension | 11.0% | 10.8% | 11.2% | 10.5% |
| Thrombocytopenia | 15.5% | 15.8% | 15.8% | 16.4% |
| Stroke | 13.7% | 14.0% | 13.4% | 12.9% |
| Trauma | 10.5% | 9.4% | 9.2% | 10.0% |
| *Exlihauser Comorbid Conditions* |  |  |  |  |
| Congestive Heart Failure | 45.8% | 45.0% | 45.7% | 46.6% |
| Cardiac Arrhythmias | 52.1% | 53.0% | 52.6% | 52.9% |
| Valvular Disease | 12.2% | 12.8% | 12.5% | 11.8% |
| Pulmonary Circulation Disorders | 16.0% | 15.5% | 16.0% | 14.0% |
| Peripheral Vascular Disorders | 11.8% | 11.9% | 12.2% | 12.3% |
| Hypertension, Uncomplicated | 40.1% | 38.7% | 39.2% | 39.4% |
| Paralysis | 9.1% | 9.2% | 8.3% | 9.2% |
| Other Neurological Disorders | 43.3% | 43.9% | 44.8% | 45.4% |
| Chronic Pulmonary Disease | 48.2% | 48.9% | 49.0% | 46.9% |
| Diabetes, Uncomplicated | 30.1% | 29.9% | 29.4% | 30.6% |
| Diabetes, Complicated | 9.0% | 9.2% | 9.1% | 8.3% |
| Hypothyroidism | 14.5% | 14.5% | 13.5% | 12.9% |
| Renal Failure | 28.8% | 30.1% | 30.3% | 31.6% |
| Liver Disease | 7.8% | 10.1% | 9.7% | 9.0% |
| Peptic Ulcer Disease Excluding Bleeding | 1.8% | 1.6% | 1.6% | 1.7% |
| AIDS/HIV | 0.5% | 0.3% | 0.3% | 0.6% |
| Lymphoma | 0.7% | 1.5% | 1.1% | 1.0% |
| Metastatic Cancer | 1.5% | 1.4% | 1.3% | 1.5% |
| Solid Tumor Without Metastasis | 3.6% | 3.8% | 3.4% | 4.0% |
| Rheumatoid Arthritis/Collagen Vascular | 4.6% | 2.9% | 3.7% | 3.5% |
| Coagulopathy | 19.3% | 20.0% | 19.7% | 20.5% |
| Obesity | 21.5% | 21.1% | 22.2% | 20.3% |
| Weight Loss | 41.0% | 43.5% | 44.4% | 42.1% |
| Fluid and Electrolyte Disorders | 76.8% | 78.2% | 80.0% | 80.1% |
| Blood Loss Anemia | 1.7% | 1.8% | 1.6% | 1.9% |
| Deficiency Anemia | 4.7% | 4.5% | 3.8% | 3.4% |
| Alcohol Abuse | 5.2% | 6.6% | 5.9% | 5.6% |
| Drug Abuse | 2.9% | 3.1% | 3.6% | 3.2% |
| Psychoses | 3.5% | 2.9% | 2.8% | 3.2% |
| Depression | 11.9% | 11.6% | 12.1% | 10.8% |
| Hypertension, Complicated | 27.4% | 28.9% | 28.0% | 29.4% |
| Average LTACH Pressure Ulcer Score | 1.6 | 1.8 | 1.8 | 1.7 |
| Average LTACH Readmission Score | 25.4 | 25.1 | 25.2 | 25.2 |
